# A century of temporal stability of genetic diversity in wild bumblebees

**DOI:** 10.1038/srep38289

**Published:** 2016-12-05

**Authors:** Kevin Maebe, Ivan Meeus, Sarah Vray, Thomas Claeys, Wouter Dekoninck, Jean-Luc Boevé, Pierre Rasmont, Guy Smagghe

**Affiliations:** 1 Department of Crop Protection, Faculty of Bioscience Engineering, Ghent University, Coupure links 653, B-9000, Ghent, Belgium; 2 Laboratoire de Zoologie, University of Mons, Place du Parc 20, B-7000 Mons, Belgium; 3 Department of Geography, University of Namur, Rue de Bruxelles 61, B-5000 Namur, Belgium; 4 Royal Belgian Institute of Natural Sciences, Vautierstraat 29, B-1000 Brussels, Belgium

## Abstract

Since the 1950s, bumblebee (*Bombus*) species are showing a clear decline worldwide. Although many plausible drivers have been hypothesized, the cause(s) of this phenomenon remain debated. Here, genetic diversity in recent *versus* historical populations of bumblebee species was investigated by selecting four currently restricted and four currently widespread species. Specimens from five locations in Belgium were genotyped at 16 microsatellite loci, comparing historical specimens (1913–1915) with recent ones (2013–2015). Surprisingly, our results showed temporal stability of genetic diversity in the restricted species. Furthermore, both historical and recent populations of restricted species showed a significantly lower genetic diversity than found in populations of co-occurring widespread species. The difference in genetic diversity between species was thus already present before the alleged recent drivers of bumblebee decline could have acted (from the 1950’s). These results suggest that the alleged drivers are not directly linked with the genetic variation of currently declining bumblebee populations. A future sampling in the entire distribution range of these species will infer if the observed link between low genetic diversity and population distribution on the Belgium scale correlates with species decline on a global scale.

One of the important variables in evolutionary biology and population genetics is the effective population size (*N*_*e*_)^1^. *N*_*e*_ is the size of an ideal population which has the same genetic diversity as the actual population of interest^2^. Due to violations of the ideal scenario (e.g. all specimens should have equal reproduction and survival probabilities), wild populations usually depart from *N*_*e*_ which is generally smaller than the corresponding census population size (*N*_*c*_)^2^. Although many factors are linked with population viability, by estimating *N*_*e*_ one can determine the viability of a population (e.g. ref. 3) and in turn the conservation status of this population or species^1^.

The most important conservation consequence of a decrease in *N*_*e*_ is that it will result in a loss of genetic diversity^2^,^4^,^5^,^6^. The presence of sufficient genetic variation is crucial for the persistence of populations, as the loss of genetic variation will lead to a lower adaptive ability in response to current and future changes in the environment, such as new pathogens, climate change and habitat loss, and can ultimately lead to extinction^4^,^5^,^6^. On a short term, populations with a low *N*_*e*_, are more vulnerable to random processes, such as genetic drift (e.g. refs 4, 5, 6). The higher effects of drift within small populations cause a (further) decrease in genetic variation. In turn, the chance of inbreeding will increase, and can lead to a decreased fitness due to inbreeding depression (e.g. refs 4, 5, 6). In social insects such as bumblebees, *N*_*e*_ will be very low in relation to the observed number of specimens, as most bumblebee species are monoandrous and their colonies consist mostly out of only one founder queen (e.g. refs 7, 8, 9).

Currently, several methods are described to estimate *N*_*e*_ in natural populations. In general, temporal studies, which estimate *N*_*e*_ by examining allele frequency changes over time, showed the best results (reviewed by refs 6,10, 11, 12). Although favourable, these approaches will be limited to only a few organisms as it requires time series, i.e. the availability of multiple specimens from populations sampled at two or more points in time (as reviewed by Habel *et al*.^6^). Although challenging, temporal sampling methods were already successfully applied to estimate *N*_*e*_ in natural populations (reviewed in ref. 6). In this manner several studies demonstrated a decrease of genetic diversity or an increase in genetic differentiation (e.g. refs 6,13,14). For instance, Athrey *et al*.^15^ compared historical and recent populations of the endangered golden-cheeked warbler (*Dendroica chrysoparia*) to assess the impact of demographic changes. Over a period of 100 years, genetic diversity decreased, while genetic differentiation increased. In contrast, other studies clearly showed temporal stability of population structures^16^,^17^,^18^. The absence of significant shifts over time could be attributed to past population dynamics which has a direct impact on genetic drift and gene flow.

In bumblebee species, which are all essential pollinators in natural and managed ecosystems (e.g. refs 9,19), new data has recently raised the debate whether the population structure remained temporally stable or whether shifts in genetic diversity occurred during recent times^20^,^21^. Many bumblebee species show major population declines in different parts of the world (e.g. refs 22, 23, 24). Specifically in Europe, 24% of the 68 European bumblebee species are threatened with extinction. More generally, bumblebee populations tend to decline (46%), remain stable (29%) or increase (13%)^25^,^26^. In populations of the restricted and currently declining bumblebee species, a lower genetic diversity than contemporary widespread and stable bumblebee species was observed^20^,^21^,^27^. So it remained unclear whether this difference is caused by the decrease in population size or whether it is an intrinsic characteristic of the currently declining species^20^,^21^,^27^. The generally accepted hypothesis says that the lower genetic diversity in the declining species is due to a reduction of genetic diversity caused by the drivers of bee decline (e.g. refs 27,28). This decline is possibly due to, or at least influenced, by (a combination of) the following drivers: pathogen infection and/or spill-over from domesticated pollinators, use of pesticides, food depletion, climate change, and landscape modifications (e.g. refs 29, 30, 31). Although land cover changed substantially during the last three millennia due to anthropogenic activity^32^, the increasing loss of habitats and forage resources due to the agricultural intensification, is thought to be the key driver of the observed bumblebee decline in Europe which started around the 1950s^24^,^30^,^33^.

Due to the impact of these drivers, the effective population size of restricted bumblebee species may decrease. Genetic drift (e.g. for changes of allele frequency in a population due to random sampling of organisms) may lead to a decrease of genetic variation within a population^4^,^5^. This stochastic loss of alleles will be more pronounced in small populations^4^,^5^, and if the population is isolated this effect may be less buffered by dispersal. This difference in vulnerability towards the impact of genetic drift will be reflected in the observed level of genetic variation, with lower and decreased levels within the populations of the restricted bumblebee species. However, this hypothesis is solely based on recent contemporary samples^22^,^27^,^28^. In contrast, studies using only historical specimens revealed similar differences in genetic diversities between declining and stable bumblebee species in North-America^20^ and Europe^21^, which suggests that no recent major reduction in genetic diversity occurred^20^,^21^. In an alternative hypothesis, populations of restricted bumblebee species have already a lower genetic diversity due to past population dynamics. These species may maintain the lower level of genetic diversity in their populations by (i) receiving enough dispersing specimens from neighboring subpopulations to counter the effects of drift, and/or (ii) as their populations may always been small, most of their genetic variation may already be removed due to selection and drift. The latter suggests that a further decrease of genetic variation will become difficult to notice over short time periods. Testing which hypothesis is causing the observed lower genetic variation in the restricted and declining species is necessary to improve conservation strategies and secure the pollination services of wild bumblebees^5^,^27^. Indeed, lower genetic diversity will predispose these populations to have a more limited ability to adapt to the changing environment^4^,^5^. For instance, genetically pauperized bumblebees are more susceptible to diseases. Research performed by Whitehorn *et al*.^34^ in the UK showed that populations of the large carder bee or moss carder bee (*B. muscorum*) with a lower level of heterozygosity showed a higher prevalence of the gut parasite *Crithidia bombi.* The same negatively correlated interaction between parasite prevalence and genetic diversity was found for the parasitic mite *Locustacarus buchneri* in *B. muscorum* but not in *B. jonellus*^35^. Furthermore, in North-America, researchers found also a link between the level of genetic diversity and an increased vulnerability to the pathogen *Nosema bombi*^22^.

In this study, we compared two groups of bumblebee species, those currently widespread and found in different parts of Belgium *versus* those currently restricted and limited to specific localities, to investigate whether a reduction in genetic diversity has occurred over time. More specifically, we compared the genetic diversity of eight bumblebee species before and after the general bee decline that started in Europe around the 1950s^35^,^36^. Pin-mounted museum specimens from 100 years ago (1913–1915) were compared to specimens collected recently (2013–2015), both sets originating from the same five locations in Belgium. As pollinator regression is intensively described in Belgium and concerns bumblebees^36^ but also other wild bees^37^,^38^, hoverflies and butterflies^36^, this country was chosen as our main study area. Among the eight chosen bumblebee species, four are currently restricted (*B. ruderarius, B. sylvarum, B. humilis* and *B. soroeensis*) and four are currently widespread (*B. pascuorum, B. hortorum, B. pratorum* and *B. lapidarius*) in Belgium. For each location and species, samples were genotyped with microsatellite DNA markers. Here, we hypothesize that (i) widespread bumblebee species have larger effective population sizes than sympatric restricted bumblebee species; (ii) genetic diversity in the potentially smaller populations of restricted species will decrease over time by the influence of genetic drift.

## Results

### Data analysis

Each of the 16 microsatellites amplified successfully in each *Bombus* species. Genotype replications for all loci were consistent, with a correct repetition of 99.71%. Based on our exclusion step of maximum 6 loci of missing values allowed within the genotype profile of a single specimen, 85 specimens were excluded from all further analyses for the historical data, and 11 for the recent ones (Table 1). Furthermore, an extra 114 and 86 specimens were removed as Colony 2.0 and Kinalyzer analyses identified them as being full-sibs within a population. Indeed, when these analyses detected a full-sib pair within one population (specimens from the same location and time period), only one random selected specimen per sibship was kept for further analysis (Table 1). After these two exclusions steps, 357 out of 566 historical and 436 out of 533 recent specimens remained in our dataset, which we used to estimate the different genetic parameters of all populations of each species (Table 1). In addition, our analyses detected no significant linkage disequilibrium between microsatellites, but found significant deviations of HW for some loci in the populations of each species. Although this difference may be due to the presence of null alleles, our analysis performed with MICROCHECKER 2.2.3 revealed only very low frequencies of null alleles (<5%) in these involved microsatellite loci.

### Estimation of genetic diversity

**Figure 1:**
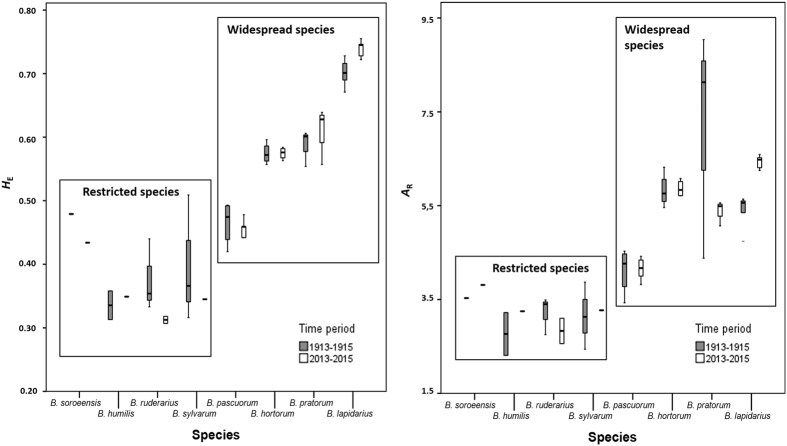
Comparison of the historical and recent genetic diversity within each *Bombus* species. Box-plots of *H*_E_ and *A*_R_ for each species and for both time periods 1913–1915 and 2013–2015.

For all *Bombus* species, the genetic diversity of all populations was estimated in the two time periods (1913–1915 and 2013–2015). Within the recent populations of the widespread *Bombus* species, the genetic diversity parameters (*A*_R_ and *H*_E_) were high, ranging from 3.820 to 6.590 and from 0.409 to 0.755, respectively (Table 2). The observed level of genetic diversity within the populations of the restricted bumblebee species was lower than within the populations of the widespread species ranging from 2.560 to 3.810 and from 0.307 to 0.434 (*A*_R_ and *H*_E_, respectively; Fig. 1).

Within the historical bumblebee populations, we found a similar result, with *A*_R_ and *H*_E_ for the widespread *Bombus* species ranging from 3.430 to 9.040 and from 0.420 to 0.728, and a lower genetic diversity within the populations of the restricted species ranging from 2.240 to 3.870 and from 0.313 to 0.509 (*A*_R_ and *H*_E_ respectively; Table 2 and Fig. 1).

### Genetic diversity in restricted versus widespread species

After running the LMM, we found no decrease of *H*_E_ over time. Indeed, the factor ‘“period” was not present in the best models (delta > 2; Table 3). Therefore, *H*_E_ remained stable over time for the restricted species with 0.385 *versus* 0.351 (mean *H*_E_ in 1913–1915 and 2013–2015, respectively) and for the widespread species with 0.589 *versus* 0.594 (mean *H*_E_ in 1913–1915 and 2013–2015, respectively; Table 2). Although, mean *A*_R_ remained fairly stable for the restricted species from 3.127 to 3.198 and for the widespread species with 5.519 *versus* 5.443 (mean *A*_R_ and *H*_E_ in 1913–1915 and 2013–2015, respectively; Table 2), time period was included in the best LMM models for *A*_R_ (delta = 0.000, Table 3). However, the effect of time period was not significant (LMM, *P* = 0.758, Table 4), neither with the interaction of “distribution” and the different “subgenera” (LMM, *P* = 0.910, and *P* = 0.088–0.802, respectively; Table 4). In general, these results show that the historical and recent genetic diversity within the populations of the restricted and widespread species did not decrease over 100 years; at least not in a consistent manner.

The models (delta < 2) fitting the observed pattern of the genetic diversity variable *H*_E_ best were the model with species “distribution” and “subgenera” as fixed factors and with or without the interaction between “distribution” and “subgenera”. The model (M12) was the best fitting model (delta = 0.000) and had the highest weight (0.632; Table 3). For the variable *A*_R_, the models with species “distribution”, “period” and “subgenera” as separate main fixed factors and with or without the interaction between “distribution” and “subgenera” and between “subgenera” and “period” had the lowest delta AIC score (Table 3). Although model (M12) showed a similar significant result for *A*_R_ (delta = 0.648, weight = 0.181; Table 3), the models including “period”, with or without the interaction of “period” with “subgenera”, were always better fitting the data (for both M40 and M48, delta = 0.000 and weight = 0.250, respectively; Table 3). Therefore, model M12 and model M48 were selected and performed as best fitting models for *H*_E_ and *A*_R_, respectively (Tables 3 and 4).

Species distribution was significantly explaining the observed pattern of *H*_E_ (LMM, *t*-test, *t* = −5.803, *p* < 0.001) and *A*_R_ (LMM, *t*-test, *t* = −3.520, *p* < 0.001) (Table 4), which means that the widespread bumblebee species had a higher genetic diversity than the restricted species, within and between both time periods. For both parameters, species subgenera had also a significantly effect on the observed pattern of genetic diversity. Indeed, compared with the other subgenera, the species of the subgenera *Melanobombus* had a higher *A*_R_ and *H*_E_ (LMM, *t*-test, *t* = 4.889, *p* < 0.001; *t* = 1.965, *p* < 0.049; respectively; Table 4) and in *Thoracobombus* a lower *H*_E_ (LMM, *t*-test, *t* = −3.348, *p* < 0.001; Table 4).

### Effective population size estimation

The effective size of each population, with data from two time points available, were measured with MLN_e_ (Table 5). Within one species our estimations of *N*_e_ varied remarkably depending on the population. Although this complexes the comparison of *N*_*e*_ between species, we can clearly distinguish the lower *N*_*e*_ in the populations of *B. sylvarum* and *B. soroeensis (N*_*e*_ = 160.6 and *N*_*e*_ = 239.0, respectively) in comparison with the *N*_*e*_ within the populations of all stable bumblebee species (Table 5). Furthermore, the estimates also showed large population sizes for *B. hortorum* (Table 5). *B. pascuorum, B. pratorum* and *B. lapidarius* showed similar values in *N*_*e*_ as those observed in *B. ruderarius* and *B. humilis* populations (Table 5), although the *B. pascuorum* population of Torgny showed a very low *N*_*e*_ *(N*_*e*_ = 219.0) comparable with the *N*_*e*_ observed in the populations of *B. sylvarum, B. soroeensis,* and *B. ruderarius* in Torgny.

## Discussion

### Lower genetic diversity in restricted species

Hundred years ago, and still today, restricted bumblebee species compared to widespread species had, and have, a significantly lower genetic diversity (Fig. 1). This result confirms those on the difference in level of genetic diversity between declined and more stable bumblebee species in The Netherlands by using only historical bumblebee specimens^21^. Hence, the here unique experimental setup of comparing recent with historical genetic diversity of different bumblebee species coming from the same locations in Belgium enabled us to show, moreover, that the levels of genetic diversity remained fairly stable over time in the studied populations. Indeed, in general, no major reduction in genetic diversity is observed over time. These results do not support the hypothesis based on solely recent specimens, which explains the difference in genetic variation between stable and declining species by a reduction of genetic diversity due to population declines in response to environmental drivers which acted around 1950 (e.g. refs 27,28). The present results rather corroborate the hypothesis that for some species (here, the restricted species) the levels of genetic diversity were already low at the beginning of the 20^th^ century, thus well before the 1950s when the agricultural revolution started with a massive use of pesticides and fertilizers. Although our results clearly support the latter hypothesis, we do not rule out the possibility that a small reduction in genetic diversity has occurred during the last 100 years. A possible and small reduction in genetic diversity may be undetectable due to the lack of statistical power and the rather small sampling sizes within the experimental setup of our study. Although we admit that an increase in sample size would be preferable to strengthen the power of the results in our study, the potential reduction would be much smaller than the here observed difference between restricted and widespread species. Furthermore, we provide here the best possible sampling and setup considering the possible difficulties when using and genotyping historical old museum and collection material. Hence, a tendency towards a small decrease in genetic diversity over time can be seen for the restricted bumblebee species, *B. ruderarius* and *B. soroeensis* (Fig. 1). However, this reduction is too small to explain the observed difference between the restricted and widespread species.

### Change of genetic diversity over time

Our results are indicative for a temporal stability of genetic diversity within the studied populations of the restricted bumblebee species. A comparable example of temporal stability of genetic diversity is shown in Danish populations of the large blue butterfly (*Maculinea arion*)^39^. Although declined the population showed no shift in genetic variation over 77 years. The authors mentioned several possible hypotheses to explain this result: i) the population decline was not strong enough to cause a reduction of genetic diversity; ii) the butterfly might decline as it relies on a host ant species*, Myrmica sabuleti*, which experiences a decline, while the butterfly’s genetic diversity is maintained by gene flow or dispersal; iii) too low statistical power in the analysis of genetic diversity due to insufficient historical sampling, or iv) the historical populations were already genetically impoverished before the start of their study. The latter explanation turned out to be the most likely one with a lower genetic variation due to long-term isolation from nearby populations^39^. Here, we can formulate some similar explanations. Although we cannot totally exclude the possibility that the population decline was not severe enough to cause a reduction of genetic diversity in bumblebee species, this seems less possible, considering the major population declines observed for most bumblebee species^24^,^30^,^33^. As described above, the low statistical power due to insufficient historical sampling may be another possibility here. Especially, if one takes into account that all comparisons between historical and recent genetic diversity levels in the restricted species were assessed from one location (Torgny; Table 2). Finally, the absence of recent specimens at the other locations for these species is already indicative of their population decline, and may also represent a non-measurable loss of genetic diversity at these locations.

Although no significant reduction of genetic diversity is detected, a significant increase of genetic diversity over time was observed for the widespread species *B. lapidarius* (subgenera *Melanobombus;* Table 5 and Fig. 1). A possible explanation of this observed effect is an increase in effective population sizes and/or a higher level of gene flow by dispersal between populations in this species.

### Other possible causes of the lower genetic diversity in the restricted bumblebee species

**Figure 2:**
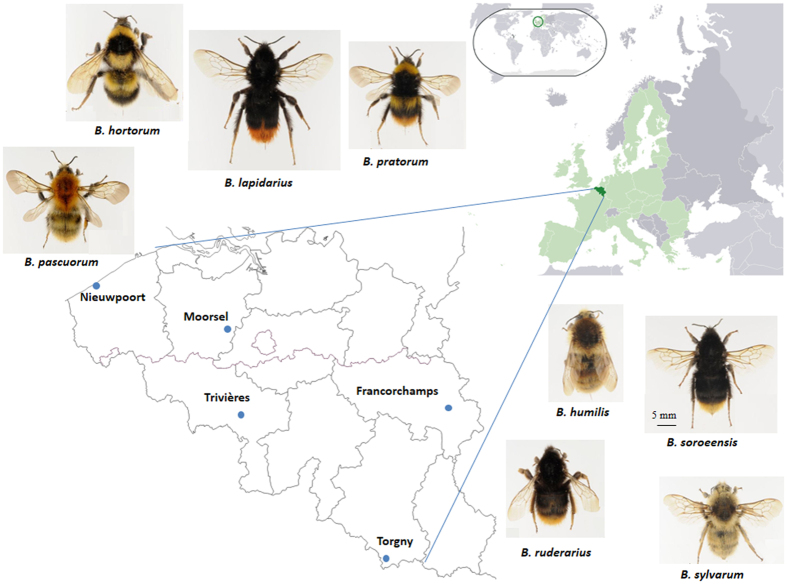
Overview of the bumblebee species sampled at five locations in Belgium. Specimens for each species were collected at the same five locations in Belgium in 1913–1915 and 2013–2015. Pictures of species are from Rasmont & Pauly^60^, and an adapted map of Belgium from http://www.d-maps.com/conditions.php?lang=en.

The question about genetic reduction now somewhat shifts. If it did not occur over the last 100 years, did it occur earlier? The low genetic profile within the historical populations of the restricted species was perhaps already altered due to genetic bottlenecks occurring even before the beginning of the 20^th^ century. In Europe, land cover use changed substantially due to anthropogenic activity, such as a large scale deforestation during the Industrial Revolution (ca. 1790–1900)^32^ which may have caused earlier bumblebee declines. In turn, most of the genetic variation could be already removed from their populations, with only the necessary adaptive genetic variation remaining^40^. The detection of an additional reduction, within the 100 years of our study, would thus become even more difficult to detect. Although having low levels of genetic variation, without a strong (new) external pressure on the environment, these populations would not be heavily affected^40^. However, once the possible drivers of bee decline acted in the 1950’s, they may have caused the trigger that started the decline and even the extinction of these species. Although a very possible explanation, but in the absence of robust collection material from the 19^th^ century, it is impossible to test this possibility, let alone the general technical difficulties in using haplodiploid species. In our study we present species belonging to different subgenera which could represent a different genetic diversity and could bias the difference observed between restricted and widespread species. Indeed, the over representing of different species of the subgenus *Thoracobombus* could bias the observed lower genetic diversity within the group of restricted bumblebee species. However, we speculate that this is not the case. Indeed, even within the subgenera *Thoracobombus*, the widespread species (*B. pascuorum*) had a significantly higher genetic diversity than the restricted species (*B. ruderarius, B. sylvarum* and *B. humilis*; Table 2 and Fig. 1). The lower levels of genetic diversity in the restricted bumblebee species may be a specific character of these species, and thus do not necessarily mean that there was a population bottleneck or decline. Many species have vast differences in their effective population sizes and genetic diversity^2^. For instance, Romiguier *et al*.^41^ revealed a strong influence of life-history traits (such as body mass, longevity, and reproductive strategy) on genetic diversity by a comparative analysis of patterns of diversity across several animals. A bumblebee species in which the mother queen produces much reproductives (daughter queens and/or males) may have a higher level of genetic diversity than a species which produces less reproductives. Whether this is effectively the case is not known. More research concerning the role of species specific characteristics on the observed difference in genetic diversity between these bumblebee species might bring more clarity. The relatively low genetic diversity of the restricted species may be explained by the smaller population sizes of these species. Indeed, restricted bumblebee populations may have smaller population sizes and thus can have a reduced genetic diversity as a result of higher genetic drift and if isolated also a reduced gene flow^4^,^6^. Although it seems that genetic drift did not result in an extra reduction over the last 100 generations, our results showed that restricted bumblebee species have lower effective population sizes. Species’ smaller distribution is thus a valid explanation of the low genetic diversity observed in the restricted species which are now more heavily affected by the drivers of the decline. Hence, the question can perhaps be rephrased: is a low *H*_E_ and *A*_R_ intrinsically linked with the biology of a certain species, or with being locally restricted? Here we defined the distribution range based on data from a small ecological scale (see Table 6 and Fig. 2). A sampling on a larger ecological scale, within the whole distribution range of these species, will allow for comparison of the same species being locally widespread and locally restricted. This will determine the link between species and an intrinsic level of genetic diversity. In turn, this will have great influence on the implementation of future conservation strategies.

## Methods

### Sampling of historical and recent specimens

Historical bumblebee specimens were collected from the Hymenoptera collection of the Royal Belgian Institute of Natural Sciences (RBINS) in Brussels. Five locations in Belgium were selected due to the presence of sufficient available historical bumblebee specimens of multiple species for genetic analysis, and since these five locations represent the main bio-geographical units of the country. In these five locations (Francorchamps, Moorsel, Nieuwpoort, Trivières, and Torgny; Fig. 2), bumblebees were collected within a 5 × 5 km^2^ frame. Two neighboring localities were merged together for Trivières (Trivières and St-Vaast) and Torgny (Torgny and Lamorteau) to allow a comparable sampling area as within the other localities. Historical specimens from the RBINS collection were collected in the bumblebee foraging season 1913, 1914, and 1915 (Table 1), while recent specimens were sampled in 2013, 2014 and 2015 at the same locations. For both historical and recent time periods, 20 to 25 specimens were selected when possible from each location for genetic analyses resulting in the selection of 566 historical and 533 recent specimens (Table 1).

All bumblebee species belong to only one genus, *Bombus*, but are divided in different subgenera (for a full division of species in subgenera, see ref. 42). The eight bumblebee species selected here belong to five different subgenera: *Kallobombus, Megabombus, Melanobombus, Pyrobombus*, and *Thoracobombus* (see Table 1)^42^.

Here, the further division of the eight bumblebee species in two groups of four currently restricted and four currently widespread species is based on three layers of available distribution or abundance data: (i) the available abundance data of these species within the Belgian collection^36^,^37^, with a clear significant difference between both groups within the two time periods by using a linear mixed model (LMM, lmer(log(abundance) ~ distribution + (1|species), data = Data) in R studio^43^ with R package lme4 version 1.1–10^44^ (LMM, *t-*test, *t* = 6.721, *p* < 0.001; Table 6); (ii) the presence and/or absence of the species at each location during the historical and recent bumblebee foraging season (Table 6): and (iii) the population trend of these species within Europe, with the group of restricted species having a “decreasing” population trend and the widespread species a “stable” or “increasing” population trend (ref. 25; Table 6).

### Bumblebee DNA extraction and microsatellite protocol

One middle leg of each individual bumblebee specimen was used for DNA extraction. DNA extraction, PCR amplification with 16 microsatellite markers (four multiplexes of four loci), and visualization with capillary electrophoreses on an ABI-3730xl sequencer (Applied Biosystems), were performed with the method as described in Maebe *et al*.^21^. The four microsatellite markers (B11, B100, B126, B132) developed by Estoup *et al*.^45^; and the four loci (BL02, BT04, BT08, and BT10) developed by Reber-Funk *et al*.^46^ were chosen based on Maebe *et al*.^21^. Of the other remaining eight markers, five loci (BL13, BT02, BT05, BT23, BT24) were developed by Reber-Funk *et al*.^46^ and three loci (0294, 0304 and 0810) by Stolle *et al*.^47^. From the 1099 genotyped specimens, 128 random selected specimens (11.65%) were re-genotyped to examine the genotypic error rate.

### Data analysis

Some of the genotyped specimens were excluded prior to data analyses, after applying several validation steps following Maebe *et al*.^21^. In short, specimens were removed when they could not be scored in a reliable manner for a minimum of 10 microsatellite loci, and only one random specimen per sibship was kept after sister identification with the programs Colony 2.0^48^ and Kinalyzer^49^. Furthermore, genotypic linkage disequilibrium, deviations from Hardy-Weinberg equilibrium (HW), and evidence of null alleles were tested using the programs FSTAT 2.9.3^50^, GENALEX 6.5^51^ and MICROCHECKER^52^, respectively.

### Estimation of genetic diversity

For each population we determined the genetic diversity based on two parameters: the allelic richness (*A*_*R*_) estimated as the sample size-corrected private allelic richness with the program HP-Rare 1.1^53^ calculated and normalized on 10 diploid specimens for all populations, and Nei’s unbiased expected heterozygosity (*H*_E_;^54^) calculated with the program GENALEX 6.5^51^.

### Comparison of genetic diversity between species groups

To examine whether genetic diversity differed between species, and/or whether other factors such as species distribution, species subgenera, locations and/or time period had an effect on genetic diversity, we conducted LMM’s in RStudio^43^. Species and sample location were chosen as random factors: (i) species, since the genetic diversity of a specific species is correlated over time and location; and (ii) location, as specimens were resampled at each location. Fixed factors were: time period (1913–1915 or 2013–2015), species subgenera (belonging to which bumblebee subgenera), and species distribution (widespread or restricted, see explanation above and in Table 6). The model that best fitted the pattern in genetic diversity was selected by using the Akaike’s Information Criterion (AIC). The MUMIn package with the dredge command allowed us to calculate all possible combinations and thus model selection^55^. As described in Maebe *et al*.^21^, problems linked with the interpretation of inter-specific differences could arise, for instance, in microsatellite mutation rates and levels of polymorphisms. Therefore, we added species as a random factor in the model. Furthermore, species belonging to the same subgenera could have comparable levels of genetic diversity due to similar mutation rates and dispersal abilities. Thus, over-representing of species belonging to one subgenera in the two groups (widespread *versus* restricted bumblebee species) could cause bias in our analyses. For instance, bumblebee species of the subgenera *Pyrobombus* may have higher dispersal abilities than *Thoracobombus* species^56^,^57^. As species with more limited dispersal rates will have less chance of a successful recolonization, they will be more vulnerable to genetic drift and thus may have less genetic variation. Thus, as division in subgenera could influence the level of genetic diversity, subgenera was included in the LMM. The best LMMs were run in R studio with R package lme4 version 1.1–10^44^.

### Effective population size

The estimation of *N*_*e*_ of each population was performed using one multiple temporal method: a maximum-likelihood approach implemented in the program MLN_e_^58^. This method recently became recommended for the estimation of *N*_*e*_ in natural populations under both ideal and migration scenarios (see ref. 59). In short, and contradicting other methods, this method successfully takes migration into account when estimating *N*_*e*_ which otherwise could bias *N*_*e*_ estimation. A generation time of 1 year was used, as most bumblebee species have one life-cycle per year.

## Additional Information

**How to cite this article**: Maebe, K. *et al*. A century of temporal stability of genetic diversity in wild bumblebees. *Sci. Rep.* **6**, 38289; doi: 10.1038/srep38289 (2016).

**Publisher's note:** Springer Nature remains neutral with regard to jurisdictional claims in published maps and institutional affiliations.

## Figures and Tables

**Table 1:** Number of specimens genotyped in the analysis categorized per *Bombus* species.

Species	Subgenera	Historical					Recent				
		Pop	n	NA	FS	*N*	Pop	n	NA	FS	*N*
*B. hortorum*	*Megabombus*	4	97	3	6	88	4	94	1	8	85
*B. humilis*	*Thoracobombus*	2	33	2	12	19	1	15	1	6	8
*B. lapidarius*	*Melanobombus*	5	100	18	13	69	5	122	3	8	111
*B. pascuorum*	*Thoracobombus*	4	101	14	20	67	5	140	0	31	109
*B. pratorum*	*Pyrobombus*	3	69	9	22	38	4	97	3	12	82
*B. ruderarius*	*Thoracobombus*	3	75	16	23	36	2	20	2	1	17
*B. soroeensis*	*Kallobombus*	1	25	12	2	11	1	21	0	12	9
*B. sylvarum*	*Thoracobombus*	3	66	11	16	39	1	24	1	8	15
*Total*		25	566	85	114	367	23	533	11	86	436

With indication of their division in *Bombus* subgenera, and with pop = the number of populations sampled, *n* = the total number of specimens genotyped, *NA* = the number of specimens that were not amplifiable, *FS* = the number of detected and removed full sibs, and N = the final number of workers used in all further analyses.

**Table 2:** Comparison of the genetic diversity within historical and recent populations of *Bombus* species.

	Species	Location	Historical time period (1913–1915)					Recent time period (2013–2015)				
			*N*	*H*_E_	SE	*A*_R_*	SE	*N*	*H*_E_	SE	*A*_R_*	SE
Restricted species	*B. soroeensis*	Torgny	11	0.479	0.079	3.530	0.514	9	0.434	0.095	3.810	0.680
	*B. humilis*	Trivières	13	0.358	0.079	3.220	0.527		—	—	—	—
	*B. humilis*	Torgny	6	0.313	0.077	2.310	0.339	8	0.349	0.080	3.250	0.556
	*B. ruderarius*	Moorsel	10	0.333	0.085	2.750	0.504		—	—	—	—
	*B. ruderarius*	Nieuwpoort		—	—	—	—	6	0.307	0.095	2.560	0.500
	*B. ruderarius*	Trivières	15	0.354	0.092	3.400	0.658		—	—	—	—
	*B. ruderarius*	Torgny	11	0.440	0.075	3.490	0.501	11	0.318	0.101	3.100	0.691
	*B. sylvarum*	Moorsel	7	0.316	0.089	2.440	0.484		—	—	—	—
	*B. sylvarum*	Trivières	14	0.366	0.078	3.130	0.566		—	—	—	—
	*B. sylvarum*	Torgny	18	0.509	0.074	3.870	0.498	15	0.345	0.089	3.270	0.720
		**Mean**	**11.7**	**0.385**	**0.068**	**3.127**	**0.496**	**9.8**	**0.351**	**0.045**	**3.198**	**0.400**
Widespread species	*B. pascuorum*	Francorchamps	17	0.458	0.093	4.120	0.673	26	0.478	0.085	4.340	0.727
	*B. pascuorum*	Moorsel	21	0.493	0.083	4.410	0.639	21	0.459	0.086	4.170	0.738
	*B. pascuorum*	Nieuwpoort		—	—	—	—	19	0.454	0.090	4.420	0.833
	*B. pascuorum*	Trivières	17	0.491	0.087	4.530	0.684	23	0.442	0.083	4.000	0.690
	*B. pascuorum*	Torgny	12	0.420	0.072	3.430	0.478	20	0.409	0.087	3.820	0.686
	*B. hortorum*	Francorchamps	18	0.596	0.087	6.320	0.952	25	0.584	0.092	6.080	0.981
	*B. hortorum*	Moorsel	24	0.557	0.094	5.800	0.911	20	0.580	0.095	5.950	0.929
	*B. hortorum*	Nieuwpoort	25	0.576	0.082	5.720	0.849		—	—	—	—
	*B. hortorum*	Trivières	21	0.568	0.088	5.460	0.883	17	0.572	0.084	5.720	0.845
	*B. hortorum*	Torgny		—	—	—	—	23	0.563	0.089	5.710	0.907
	*B. pratorum*	Francorchamps	18	0.554	0.081	9.040	0.686	22	0.557	0.078	5.070	0.751
	*B. pratorum*	Moorsel	12	0.606	0.061	4.380	0.568	19	0.630	0.071	5.560	0.764
	*B. pratorum*	Trivières	8	0.601	0.076	8.130	0.539	21	0.626	0.076	5.500	0.726
	*B. pratorum*	Torgny		—	—	—	—	20	0.639	0.075	5.480	0.652
	*B. lapidarius*	Francorchamps	16	0.728	0.040	5.600	0.413	23	0.722	0.056	6.250	0.546
	*B. lapidarius*	Moorsel	14	0.690	0.040	5.350	0.506	20	0.728	0.054	6.310	0.519
	*B. lapidarius*	Nieuwpoort	15	0.716	0.039	5.640	0.534	24	0.755	0.055	6.590	0.584
	*B. lapidarius*	Trivières	8	0.701	0.059	4.810	0.567	22	0.746	0.047	6.520	0.534
	*B. lapidarius*	Torgny	16	0.671	0.052	5.560	0.564	22	0.745	0.052	6.480	0.643
		**Mean**	**16.4**	**0.589**	**0.091**	**5.519**	**1.377**	**21.5**	**0.594**	**0.111**	**5.443**	**0.898**

For each population the mean values (and SE) of the expected heterozygosity (*H*_E_) and the allelic richness (*A*_R_) over all microsatellite loci are given. Furthermore, species are grouped based on their distribution in Belgium. With *N* = the number of populations of each species.

^*^Allelic richness calculated based on 10 diploid specimens.

**Table 3:** Selection of best fitting model explaining the genetic diversity in *Bombus*.

A	*H*_E_	(Intercept)	Distribution	Subgenera	Period	Distribution: Subgenera	Distribution: Period	Subgenera: Period	df	logLik	AIC	delta	weight
	M4	0.554	+	+	*NA*	*NA*	*NA*	*NA*	9	71.762	−120.79	0.000	0.496
	M12	0.554	+	+	*NA*	+	*NA*	*NA*	9	71.762	−120.79	0.000	0.496
	M8	0.584	+	+	+	*NA*	*NA*	*NA*	10	68.195	−110.44	10.343	0.003
**B**	***A***_**R**_	**(Intercept)**	**Distribution**	**Subgenera**	**Period**	**Distribution: Subgenera**	**Distribution: Period**	**Subgenera: Period**	**df**	**logLik**	**AIC**	**delta**	**weight**
	M40	4.878	+	+	+	*NA*	*NA*	+	14	−45.151	131.030	0.000	0.250
	M48	4.878	+	+	+	+	*NA*	+	14	−45.151	131.030	0.000	0.250
	M4	4.742	+	+	*NA*	*NA*	*NA*	*NA*	9	−54.470	131.678	0.648	0.181
	M12	4.742	+	+	*NA*	+	*NA*	*NA*	9	−54.470	131.678	0.648	0.181
	M56	4.915	+	+	+	*NA*	+	+	15	−44.719	134.438	3.408	0.046

Of all possible models run under MUMIn^55^ using species distribution, species subgenera, location and both time periods as fixed effects and species as a random effect, the best fitting linear mixed-effect models (with a delta < 4) are given. The final selected models for A. *H*_E_ and B. *A*_R_ were indicated in bold following their high (negative or positive) Akaike’s Information Criterion (AIC) and weight of fitting the pattern. With + = parameters included in the model, and NA = not included parameters.

**Table 4:** Output of the selected linear mixed - effect models (LMM).

A.	*H*_E_	Estimate	SE	*t*-value	*p*
	Distribution	−0.098	0.017	−5.803	<**0.001**
	Megabombus	0.013	0.035	0.371	0.710
	Melanobombus	0.166	0.034	4.889	<**0.001**
	Pyrobombus	0.048	0.035	1.370	0.171
	Thoracobombus	−0.098	0.029	−3.348	**<0.001**
**B.**	***A***_**R**_	**Estimate**	**SE**	***t*****-value**	***p***
	Distribution	−1.067	0.303	−3.520	**<0.001**
	Period	−0.280	0.940	−0.298	0.766
	*Megabombus*	0.988	0.803	1.229	0.219
	*Melanobombus*	1.552	0.790	1.965	**0.049**
	*Pyrobombus*	0.525	0.803	0.654	0.513
	*Thoracobombus*	−0.745	0.729	−1.031	0.302
	Period * Megabombus	0.239	1.052	0.228	0.820
	Period * Melanobombus	−0.758	1.030	−0.736	0.462
	Period * Pyrobombus	2.061	1.069	1.927	0.054
	Period * Thoracobombus	0.291	0.988	0.294	0.768

Impact of the different factors in the models on A. *H*_E_ and B. A_R_. With the estimate, standard error (SE) and *p*-value of each factor or interaction in the model obtained by *t*-tests. Significant factors are indicated in bold.

**Table 5:** Estimation of the effective population sizes (*N*_e_) with different temporal methods.

Species	Location	LMNe	95% CI
*B. soroeensis*	Torgny	**239.0**	167.6–354.0
*B. humilis*	Torgny	**1343.7**	467.7–∞
*B. ruderarius*	Torgny	**455.8**	266.8–918.1
*B. sylvarum*	Torgny	**160.6**	121.1–214.0
*B. pascuorum*	Francorchamps	**1971.0**	1096.5–5041.6
*B. pascuorum*	Moorsel	**984.6**	627.23–1730.0
*B. pascuorum*	Trivières	**1615.0**	875.2–4315.0
*B. pascuorum*	Torgny	**219.0**	167.9–288.3
*B. hortorum*	Francorchamps	**∞**	11493.0–∞
*B. hortorum*	Moorsel	**∞**	∞–∞
*B. hortorum*	Trivières	**4896.4**	17313.0–∞
*B. pratorum*	Francorchamps	**449.9**	339.6–607.1
*B. pratorum*	Moorsel	**396.1**	299.2–538.5
*B. pratorum*	Trivières	**503.1**	364.5–726.4
*B. lapidarius*	Francorchamps	**939.8**	656.9–1446.5
*B. lapidarius*	Moorsel	**658.1**	477.7–955.2
*B. lapidarius*	Nieuwpoort	**840.4**	610.5–1216.5
*B. lapidarius*	Trivières	**2851.4**	1310.2–∞
*B. lapidarius*	Torgny	**599.9**	453.5–816.6

For each method, the 95% confidence interval (CI) of *N*_e_ is given. The harmonic mean is calculated over all temporal methods for each population and over all populations of each species.

**Table 6:** Distribution and abundance data of the different bumblebee species.

Species	European Population Trend^1^	Belgian collection data^2^		Francorchamps		Moorsel		Nieuwpoort		Torgny		Trivières		N	
		<1950	>1950	T1	T2	T1	T2	T1	T2	T1	T2	T1	T2	T1	T2
*B. soroeensis*	Decreasing	526	49	+	+	−	−	−	−	+	+	+	−	3	2
*B. humilis*	Decreasing	857	27	+	−	+	−	−	−	+	+	+	−	4	1
*B. ruderarius*	Decreasing	1599	185	+	−	+	−	+	+	+	+	+	−	5	2
*B. sylvarum*	Decreasing	622	35	+	−	+	−	−	−	+	+	−	−	3	1
*B. pascuorum*	Increasing	20176	3995	+	+	+	+	+	+	+	+	+	+	5	5
*B. hortorum*	Stable	5529	865	+	+	+	+	+	+	+	+	+	+	5	5
*B. pratorum*	Increasing	3603	3597	+	+	+	+	+	+	+	+	−	+	4	5
*B. lapidarius*	Increasing	10714	971	+	+	+	+	+	+	+	+	+	+	5	5

The division of the selected *Bombus* species in restricted and widespread species is based on three sets of available data: (i) the species population trend in Europe^25^; (ii) the number of bumblebee specimens within the RBINS collection before 1950 and between 1955–1993^36^,^37^; and (iii) the presence or absence of the species at each location during the historical and recent bumblebee foraging season 1913–1915 (=T1) and 2013–2015 (=T2), respectively. With N the total number of locations where a species was found, and with V = the presence and – = the absence of the species at that specific location.

^1^Data from^25^.

^2^Data from^36^,^37^.
